# Exploring Rehabilitation Professionals' Attitudes Toward Telerehabilitation Using Developing a New Questionnaire: A Cross ‐Sectional Study

**DOI:** 10.1002/hsr2.73121

**Published:** 2026-08-31

**Authors:** Seyede Zohre, Elham Tavanai, Fatemeh Salmani, Mahdieh Karimi, Vida Rahimi

**Affiliations:** ^1^ Department of Audiology, School of Rehabilitation Shahid Beheshti University of Medical Sciences Tehran Iran; ^2^ Department of Audiology, School of Rehabilitation Tehran University of Medical Sciences Tehran Iran; ^3^ Department of Epidemiology and Biostatistics, School of Health, Geriatric Health Research Center Birjand University of Medical Sciences Birjand Iran; ^4^ Department of Speech Therapy, School of Rehabilitation Tehran University of Medical Sciences Tehran Iran

**Keywords:** attitude, professionals, rehabilitation specialists, telemedicine, telerehabilitation (TR), validity

## Abstract

**Background:**

Telerehabilitation (TR) facilitates remote access to rehabilitation services, with its growth driven by advances in internet and communication technologies. However, its implementation may be influenced by professionals' attitudes. This study aimed to develop and validate a questionnaire to assess rehabilitation professionals' attitudes toward TR and to examine the association of demographic and professional characteristics with these attitudes.

**Methods:**

This two‐phase cross‐sectional study involved 139 rehabilitation professionals from audiology, speech therapy, occupational therapy, and physiotherapy. In Phase 1, questionnaire development included face and content validity assessment by an expert panel. Exploratory factor analysis (EFA) identified the underlying structure, and confirmatory factor analysis (CFA) provided preliminary support for the model. Reliability was assessed using Cronbach's alpha. In Phase 2, the validated questionnaire was administered, and data were analyzed using independent t‐tests and one‐way ANOVA.

**Results:**

The 33‐item questionnaire demonstrated excellent validity (CVI > 0.90) and reliability (Cronbach's *α* = 0.91). EFA identified a three‐factor structure explaining 76.17% of the total variance. Participants reported generally positive attitudes toward TR, particularly regarding its potential to expand access to services across wider geographical areas and support service delivery during pandemics. Consultation was perceived as the rehabilitation process most amenable to TR. Key perceived barriers included limited access to internet and communication technologies, concerns about service quality, and service undervaluation. Attitudes did not differ significantly according to age, educational level, work experience, or clinical field. Level of knowledge significantly affected one attitude domain (*p* < 0.01), with a non‐linear pattern observed across knowledge levels.

**Conclusion:**

The developed questionnaire demonstrated satisfactory psychometric properties for assessing rehabilitation professionals' attitudes toward TR. Rehabilitation professionals generally recognized the benefits of TR, particularly its potential to improve service accessibility and continuity of care, while also identifying important barriers related to infrastructure and service quality. The questionnaire provides a comprehensive tool for evaluating attitudes toward TR and may support future research on factors influencing its adoption across rehabilitation settings.

## 1 Background

Telehealth, the remote delivery of healthcare, has grown popular for improving access, especially for those without traditional services [1]. Limited access arises from geographical, economic barriers [2], climate conditions [3], age, and physical health limitations [4, 5]. Though telehealth began in the late 19th century [6], its recent expansion has been driven by the widespread use of the internet and communication technologies [3, 7]. Advances have expanded telehealth globally, transforming it beyond patient care to include education, information sharing, and consultations [8]. According to the World Health Organization (WHO), telerehabilitation (TR) is the provision of diagnostic, therapeutic, and preventive services using communication technologies by relevant professionals from a distance [9].

TR is a recent advancement using information and communication technology to extend rehabilitation services beyond traditional limits, first introduced by the U.S. National Institute on Disability and Rehabilitation Research in 1997 [10].

The importance of TR became more apparent after the COVID‐19 pandemic, emphasizing the need to shift to telehealth to maintain care while following social distancing. Rehabilitation professionals adapted with different levels of readiness and acceptance. However, the need for TR extends beyond the pandemic [11].

The role of TR in health systems has been considered across many disciplines. Studies have investigated its effectiveness and potential benefits in various rehabilitation fields, including speech therapy, audiology, physiotherapy, and occupational therapy, and in diverse geographical locations such as England [12], Nigeria [13], Saudi Arabia [14], India [15], and Pakistan [16]. For example, a 2022 study by Nazreen Nihara et al. found that the COVID‐19 pandemic accelerated the adoption of telemedicine in audiology. While audiologists showed good knowledge and positive attitudes toward teletraining, they hesitated to use it due to practical challenges. The study highlights a gap between awareness and actual use, emphasizing the need for more training and support to fully integrate teleaudiology into practice [15]. By addressing the challenges and providing adequate resources, the field of audiology can better leverage remote technologies to improve patient care and accessibility. Researchers recommend ongoing education and professional development in telehealth for audiologists to bridge the gap between knowledge and implementation in teleaudiology services [15, 17, 18]. In the field of speech therapy, Bajaj et al. (2022) conducted a study with the aim of collecting the knowledge, attitude, and practice of speech therapists in India regarding TR services during the pandemic and stated that many of them lacked technical knowledge and required skills. They did not feel prepared to deliver TR services. In addition, most participants reported that patients were relatively less motivated and less satisfied with TR services [19]. Similarly, in the field of physiotherapy, Aloyuni et al. (2020) found that although knowledge of TR‐based physiotherapy was adequate, technical barriers and insufficient staff skills limited the implementation of this approach in physiotherapy settings [20].

Despite TR's benefits, challenges remain, especially the lack of awareness or negative attitudes among professionals. This issue is more pronounced in communities without standardized TR training programs for practitioners [21].

TR can offer benefits to both patients and professionals. For patients, it may reduce travel time and costs as well as improve access to healthcare services. For professionals, it can provide a new opportunity to offer healthcare services, allowing them to reach a wider target population and potentially improve patient outcomes. Despite the increasing interest in TR, previous studies have been limited by methodological flaws. Specifically, many studies have used questionnaires or checklists to investigate knowledge, attitudes, and barriers related to TR; however, these instruments have been criticized for lacking adequate validation and exploratory item analysis. Additionally, the domains assessed were often limited to specific areas, failing to provide a comprehensive view of the complex issues surrounding TR, and many instruments were not designed to be applicable across all rehabilitation disciplines [22, 23].

Therefore, it is crucial to understand professionals' attitudes toward telerehabilitation, especially in developing countries, where some practitioners still prefer traditional methods and are hesitant to adopt new approaches.

The purpose of this study was to develop and validate a questionnaire to assess the attitudes of professionals from various rehabilitation disciplines toward TR. In addition to questionnaire development, this study compares the attitudes of professionals across different rehabilitation disciplines based on factors such as age, gender, work experience, and education level that were not mentioned in previous studies. The findings, facilitated by a validated questionnaire, could offer valuable insights into the attitudes of rehabilitation professionals and highlight challenges and barriers to successful TR implementation.

## 2 Materials and Methods

This two‐phase study comprised questionnaire development (Phase 1), followed by implementation and analysis of rehabilitation experts' attitudes toward TR (Phase 2). To ensure validity, specific validation measures were implemented, including analysis of response distributions across demographic subgroups after administration to rehabilitation professionals. The development and validation process of the questionnaire was conducted in accordance with the Consensus‐based Standards for the selection of Health Measurement Instruments (COSMIN) guidelines [24].

### 2.1 Questionnaire Development

#### 2.1.1 Item Generation

The research process commenced with the production of the study's primary question, specifically investigating rehabilitation professionals' attitudes toward TR. Relevant information required to test this hypothesis was identified through a search of electronic library databases, leading to the preliminary pool of 100 items intended for measuring attitudes toward telerehabilitation [14]. These items were then organized into a preliminary questionnaire format. A psychometric evaluation phase followed. Utilizing a purposeful maximum variation sampling strategy, researchers selected and interviewed domain experts deemed most capable of providing valuable insights. Accordingly, the questionnaire was evaluated by an expert panel to establish validity [25]. The panel evaluated the content validity of the item pool. Additionally, panel members were invited to provide suggestions on the drafted questions and to pose open‐ended questions if they considered any further items necessary. The expert panel consisted of university faculty members from the fields of physiotherapy, audiology, speech therapy, and occupational therapy.

#### 2.1.2 Face and Content Validity Assessment

Face validity index (FVI), which is sometimes considered an initial aspect of content validity, was evaluated after the development of the measurement instrument. It involves a subjective evaluation of whether the tool appears to measure the intended construct. On the other hand, content validity pertains to the extent to which a questionnaire addresses all crucial aspects of the variable in question. Increasing the number of items in a questionnaire leads to a stronger assessment, as it enhances reliability and provides a more comprehensive measurement of the target construct [26]. Content validity offers a deeper understanding of the concept domain associated with the variable. According to Lawshe's content validity model, the selected questionnaire items were evaluated by a panel of 10 experts. Each expert was asked to assess the necessity of each item in measuring the intended construct. They rated each item using the following three categories: “Essential,” “Useful but not essential,” and “Not necessary.” These responses were then coded for further analysis. For each question, content validity ratio (CVR) was calculated. Based on Lawshe's table, the minimum acceptable CVR value for a panel of 10 experts was 0.62 [27, 28]. Questions with CVR values lower than this threshold were removed. After CVR had been calculated, the content validity index (CVI) was used to determine the relevance of the retained items to the construct being measured. CVI examines the degree of agreement between raters regarding the relevance of an item. The questionnaire was again provided to the experts, who rated the relevance of each item on a 4‐point scale: 1 = not relevant, 2 = somewhat relevant, 3 = relevant and 4 = highly relevant. CVI was calculated at both the item level and the scale level. The scale‐level CVI based on the average method (S‐CVI/Ave) was obtained by averaging the I‐CVI values across all items. The minimum acceptable value for I‐CVI is 0.78, and for S‐CVI/Avg 0.9, is acceptable. In addition, FVI was used to assess the proportion of experts who judged the questionnaire items to be simple and clear [29].

### 2.2 Psychometric Validation

#### 2.2.1 Exploratory Factor Analysis

All 34 questions were entered into the analysis at this stage. A total of 139 participants completed the questionnaire. It was necessary for the sample adequacy, as indicated by the Kaiser‐Meyer‐Olkin measure (KMO), to surpass 0.67. Exploratory factor analysis (EFA) utilized principal component analysis with Varimax rotation, focusing on eigenvalues greater than 1 and the scree plot with 3 components. The Bartlett's test rejected the assumption of no relationship between the questions [30, 31]. Ultimately, 33 items were finalized and retained in the questionnaire. The first component covered 16.05%, the second component covered 20.12%, and the third component covered 40% of the total variance.

#### 2.2.2 Confirmatory Factor Analysis

The confirmatory factor analysis (CFA) was conducted using the maximum likelihood (ML) estimation method. Various metrics were used to evaluate goodness‐of‐fit. Hu and Bentler recommended using several indices and their corresponding cut‐off values to assess data: the Tucker–Lewis Index (TLI) with a cut‐off of TLI ≥ 0.90, the Comparative Fit Index (CFI) with a cut‐off of CFI ≥ 0.90, and the Root Mean Squared Error of Approximation (RMSEA) with a cut‐off of less than 0.08 [32]. These indicators were chosen to provide a balanced view of both the overall and relative model fit. All computed values are presented in the results section.

#### 2.2.3 Reliability Assessment

The reliability of the data was assessed using Cronbach's alpha through the internal consistency method. A value exceeding 0.70 suggests reliable data [33].

#### 2.2.4 Participants

The participants in this study were graduates in audiology, speech therapy, occupational therapy, and physiotherapy, who were between 20 and 55 years old. They had clinical experience from public or private institutions across the country.

They were recruited through snowball sampling via social media platforms, including WhatsApp, Telegram, and Facebook, and the survey link was distributed to relevant professional groups [7]. Before answering the questions, participants completed an online informed consent form. Each participant was allowed to submit only one response set, which was monitored using IP‐address tracking within the survey software. Respondents were unable to modify their responses after submission. No personal or identifiable information was collected.

This survey included two parts: questions related to the demographic section and 33 questions related to the questionnaire in the initial stage, which the participants had to score from 1 to 5. In the context of the questionnaire, responses to questions 1 and 2 were rated on a scale where 1 indicated “not at all acceptable” and 5 indicated “completely acceptable.” For questions 3 to 7, the scale ranged from 1, “not at all effective,” to 5, “completely effective.” Questions 8 to 15 were rated from 1, “no role,” to 5, “essential role.” Questions 16 to 18 used a scale from 1, “no impact,” to 5, “highest impact.” Regarding question 19, 1 represented “not at all agree,” and 5 signified “very much agree.” Questions 20 to 24 were rated from 1, “not at all beneficial,” to 5, “most beneficial.” Finally, questions 25 to 33 were rated from 1, “no role,” to 5, “essential role.”

The survey was created online and made available through digital platforms for eligible professionals who wished to participate. This questionnaire took approximately 7 min to complete.

### 2.3 Ethical Approval and Informed Consent Statements

This study has been approved with code: IR.TUMS.FNM.REC.1402.032 in the Ethics Committees of Tehran University of Medical Sciences.

### 2.4 Statistical Analysis

Quantitative data were characterized by calculating measures of central tendency and dispersion, while qualitative variables were represented using frequency counts and percentages. The relationship between quantitative variables was assessed using correlation coefficient tests. The follow‐up test exploratory factor analysis was performed. For the comparison between two groups, the independent *t*‐test was used, and for the comparison between three or more groups, the one‐way ANOVA test was used. Also, Post hoc analyses were performed using Tukey's HSD test in SPSS version 22. A significance level of *p* ≤ 0.05 was used to determine statistical significance.

## 3 Results

### 3.1 Item Generation Results

38 items were included in the questionnaire for CVR examination, and 34 items were selected after CVR calculation. Regarding individual expert panel comments, some of the items suggested overlapped conceptually with items that were already included in the questionnaire, indicating agreement between the experts and the research team regarding the relevance of these items. The content of these suggestions was incorporated into the final wording of a few questionnaire items.

### 3.2 Content Validity and Reliability

Initially, the questionnaire consisted of 38 items. Following expert panel review, four items with CVR values below 0.62 were excluded. Consequently, 34 items remained and were included in the subsequent analysis, with CVR recalculated. In the second round of expert panel review, the CVR values for Q4, Q5, Q6, Q7, Q13, Q21, Q22, Q26, and Q27 were 0.80, whereas the CVR values for the remaining items were 1.00. According to Lawshe's table, these scores were acceptable for 10 experts. Regarding the CVI, the initial review by the expert panel involved suggesting minor grammatical corrections. These revisions were then re‐evaluated by the panel, and the CVI scores were calculated. All items had a CVI of 1.00 except Q1–Q3, Q6, Q7, Q9, Q10, Q12, and Q19, which scored 0.90. Upon assessing the factor loading, one item with a loading below 0.3 (Q8 = 0.27) was eliminated, resulting in a final set of 33 items for further reliability analysis, including the calculation of Cronbach's alpha. The 33‐item questionnaire had a Cronbach's alpha of 0.9 for reliability, and the FVI for all items was 1.

### 3.3 Construct Validity

**FIGURE 1:**
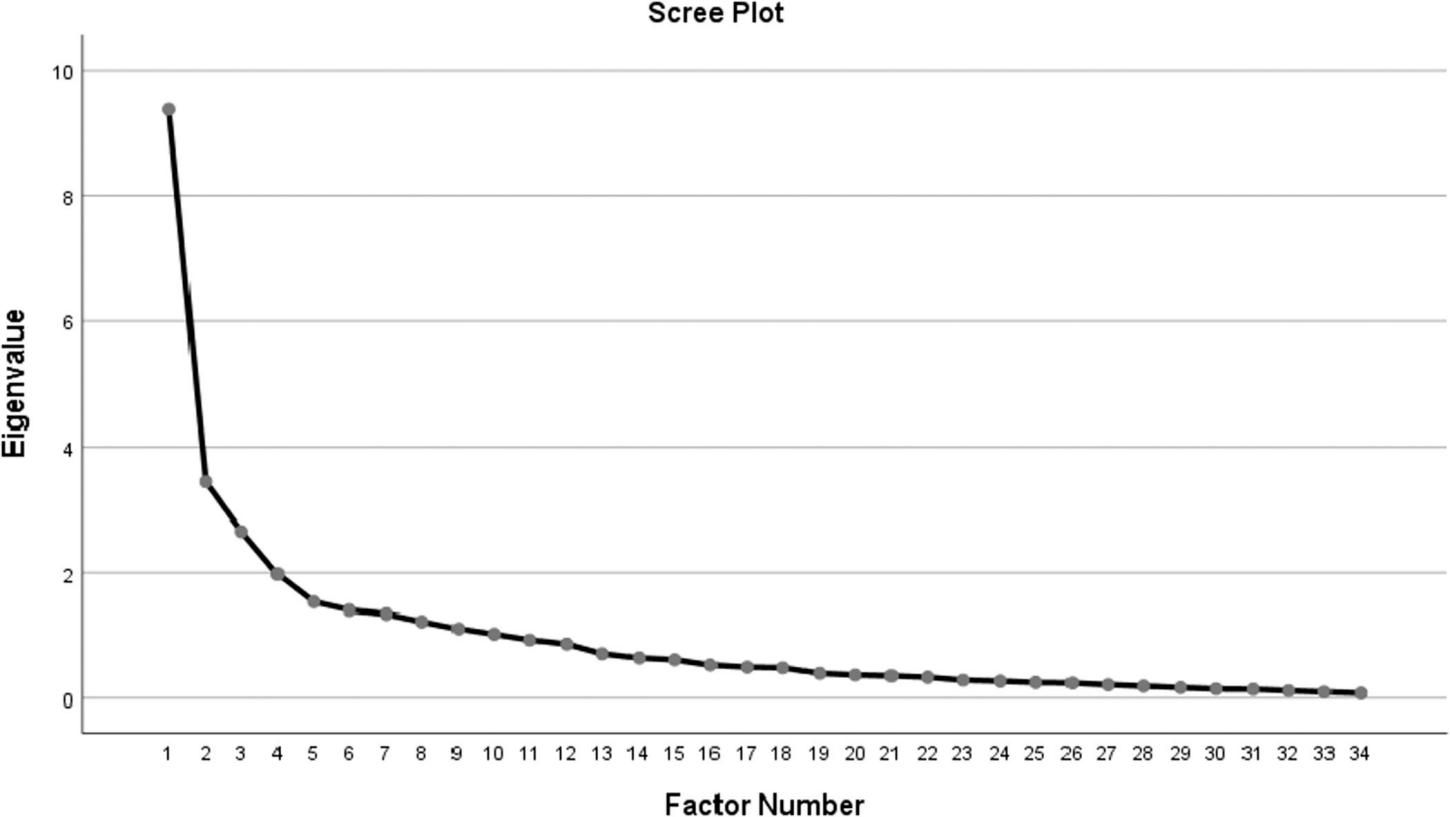
Scree plot of loading factors of 34 items.

All 34 questions were entered into the analysis at this stage. The assumption of sample adequacy was confirmed using the KMO measure of 0.794. The Bartlett's test rejected the assumption of no relationship between the questions. The scree plot (Figure 1) was examined, and with three components and considering eigenvalues greater than 1, a solution was considered. These three components were labeled “Acceptance, Effectiveness, and Role,” “Benefits and Adoption,” and “Challenges and Barriers.” The Acceptance, Effectiveness & Role component covered 20.12%, the Benefits & Adoption component covered 16.05%, and the Challenges and Barriers' component covered 40% of the total variance.

34 questions were included in the study. Items with factor loading below 0.3 were removed. This resulted in the deletion of one question (in the first version was Q8: “In your opinion, what is the role of Optometry in telerehabilitation services?”), which had a loading factor of 0.26. To maintain clarity in the final questionnaire, the subsequent questions were renumbered. Therefore, the item listed as Q8 in the final version (see Supplementary 1 and 2) corresponds to the original Q9 (“…role of Audiology…”). Furthermore, Question 11(“In your opinion, what is the role of speech therapy in telerehabilitation services?”) initially showed a cross‐loading, with its highest loading on ‘Benefits & Adoption at 0.51. However, based on its conceptual alignment with the items in the ‘Acceptance, Effectiveness & Role domain, a theoretical decision was made to reassign it. In the final rotated solution, it loaded acceptably onto Acceptance, Effectiveness & Role with a factor loading of 0.41 (see Supplementary 1). Following these adjustments, the final questionnaire comprised 33 items.

### 3.4 Confirmatory Factor Analysis

As mentioned, CFA was conducted using the ML estimation method. According to Kline's (2016) guidelines, univariate normality concerns arise when absolute skewness values exceed 3 or kurtosis values surpass 10, with values above 20 indicating significant non‐normality [34]. Following these criteria, the skewness and kurtosis indices for all items in the current study were evaluated. The results confirm that all variables remained within the acceptable thresholds of |Skewness| < 3 and |Kurtosis| < 10, indicating no major violations of normality assumptions.

The resulting fit indices—specifically Chisq/df = 1.79, PNFI = 0.64, RMSEA = 0.078, CFI = 0.90, and PCFI = 0.75 offered support for the model in accordance with established benchmarks. While the TLI (0.83) did not reach the conventional 0.90 threshold, the collective fit indices suggest a reasonable, albeit preliminary, alignment with the hypothesized structure.

### 3.5 Reliability

The final questionnaire, consisting of 33 questions, was assessed for reliability using data from all 139 participants. It demonstrated satisfactory reliability. A significant and robust relationship was noted among the various components of the questionnaire (*p* < 0.001) (Table 1).

**TABLE 1:** Final reliability and association of the domains with overall score (*n* = 139).

Dimension	Cronbach's alpha	Mean ± SD	Correlation	
			D1	D2
1:. The Acceptance, Effectiveness & Role	0.88	41.81 ± 9.34	1	
2:. Benefits & Adoption	0.88	42.15 ± 6.37	0.55**	1
3:. Challenges and Barriers	0.83	31.86 ± 6.39	0.24**	0.40**
Total	0.91	115.82 ± 17.19		

** Significant level < 0.001.

### 3.6 Population Descriptions

A total of 139 participants entered the study, and 67.7% were older than 30 years. 74.8% (104 people) were women. Generally, they had less than a doctoral degree (75.5%), and they had less than 15 years of work experience (68.3%). Most participants in this study served all three age groups (children, adults, and elderly). Most participants estimated their familiarity and use with TR at levels of “Very familiar, regular user,” “Familiar, occasional user,” “moderately familiar, rare user,” “Slightly Familiar, never use,” “Not familiar at all and do not use” (Table 2).

**TABLE 2:** Demographic characteristics of participants.

Variables		*n*	Percent
**Age**	20–25	28	20.1
	26–30	17	12.2
	31–35	24	17.3
	36–40	24	17.3
	41–50	31	22.3
	51–55	15	10.8
**Gender**	Men	35	25.2
	Women	104	74.8
**Education level**	Undergraduate	54	38.8
	Master	51	36.7
	PhD	34	24.5
**Field**	Audiologists	41	29.5
	Speech Therapy	40	28.8
	Physiotherapy	30	21.6
	occupational therapy	28	20.1
**Work experience (years)**	<5	36	25.9
	5–10	26	18.7
	11–15	33	23.7
	16–20	14	10.1
	21–25	13	9.4
	26–30	7	5.0
	>31	10	7.2
**TR experience**	Yes	76	54.7
	No	63	45.3
**Service recipient group**	Children only	47	33.8
	Adults only	28	20.1
	Elderly only	3	2.2
	All groups	61	43.9
**Knowledge and use**	1**:**Very familiar, regular user	22	15.8
	2: Familiar, occasional user	46	33.1
	3: moderately familiar, rare user	15	10.8
	4:Slightly Familiar, never use	43	30.9
	5: Not familiar at all and do not use	13	9.4

### 3.7 Distribution of Response to Attitude to TR Among Rehabilitation Professional

The distribution of participants' answers to questions in “Acceptance, Effectiveness & Role,” “Benefits & Adoption” and “Challenges and Barriers” domains is shown in Supplementary 2.

#### 3.7.1 The Distribution of Participants' Responses to Acceptance, Effectiveness & Role Domain Questions

The findings indicate that the majority of participants rated the acceptability of TR from both expert and general perspectives as somewhat acceptable (score of 2) and moderately acceptable (score of 3). These ratings were comparable concerning perceived effectiveness. However, in both domains—acceptability and effectiveness—participants assigned higher scores, indicating lesser effectiveness (score of 1) from the perspectives of the general public compared to expert assessments. In evaluating the efficiency of various communication modalities employed in TR, most participants rated audio and video communication as moderately effective (score of 3) to mostly effective (score of 4). Conversely, ratings for text‐based communication were significantly lower, falling between Somewhat effective (score of 2) and moderately effective (score of 3). Regarding the contribution of different rehabilitation disciplines in service delivery, the majority of responses indicated that these contributions ranged from minor roles (score of 2) to moderate roles (score of 3). Notably, physiotherapy and occupational therapy received higher ratings, ranging from a moderate role to a significant role (score = 4). Speech therapy was rated even higher, with scores reflecting a significant role to an essential role (score = 5).

Furthermore, concerning TR's roles in evaluation, diagnosis, and intervention across various domains, more than 50% of participants assigned scores of moderate (score of 3) or significant (score of 4) roles across all assessed areas.

#### 3.7.2 The Distribution of Participants' Responses to the Benefits & Adoption Domain Questions

In the exploratory phase, factor analysis indicated that the counseling‐related item was primarily associated with the Benefits & Adoption domain. The response distribution further demonstrated that 86% of participants rated TR as having a significant or essential role. When queried about the impact of technological advancements on future TR utilization, 88.5% of respondents attributed a significant or most significant impact to this factor. Similarly, the effects of knowledge acquisition and the mitigation of obstacles such as internet access and communication technologies—elicited comparable responses, with 82.1% and 84.1% respectively rating these as significantly impactful. Interestingly, regarding the influence of time on TR utilization, 74.1% of participants agreed or strongly agreed that the passage of time exerts no significant effect, suggesting a perception that factors like technological progress and knowledge enhancement are more influential than temporal considerations in shaping TR engagement.

Regarding the perceived advantages of TR, participants predominantly rated the benefit of reduced service delivery time as moderate (score of 3) to highly beneficial (score of 5). The advantages related to the reduction of costs and improved access for patients with special needs were mainly rated between very beneficial (score of 4) and most beneficial. Notably, a majority of participants (61.2% and 61.9%, respectively) rated the potential to provide services over larger geographical areas and during epidemics as the most beneficial.

#### 3.7.3 The Distribution of Participants' Responses to the Challenges and Barriers

In the domain addressing challenges and barriers to TR, approximately 68% of participants rated the lack of internet access by clinicians and therapists as a significant or essential obstacle. Other barriers, including limited access to information and communication technologies (ICT), insufficient knowledge among providers and patients, deterioration in service quality, concerns over automation replacing human interaction, underestimation of services from patients' perspectives, and distrust in technology, were predominantly rated as moderate to essential. The perceived impact of costs was viewed as less significant, ranging from minor to substantial. Regarding concerns about confidentiality, responses were more heterogeneous, with approximately 62% of participants assigning moderate to significant roles to these issues, underscoring differing perceptions of privacy and trust in TR systems.

### 3.8 The Effect of Age, Gender, Education Level, Work Experience, TR Experience, Service Recipient Group, Knowledge Level

#### 3.8.1 The Effect of Age on Domain Scores of the Attitude Questionnaire

Regarding age, the One‐way ANOVA analysis showed no significant differences between different age groups in the Acceptance, Effectiveness & Role (F (5133) = 0.42, *p* = 0.81), the Benefits & Adoption (F(5133) = 0.41, *p* = 0.83), Challenges and Barriers (F(5133) = 1.45, *p* = 0.20) domain scores.

#### 3.8.2 The Effect of Gender on Domain Scores of the Attitude Questionnaire

Independent *t*‐test analysis showed no significant differences between men and women participants in the Benefits & Adoption (*t* = 0.97, *p* = 0.33) and Challenges and Barriers (*t* = −0.34, *p* = 0.73). However, this difference in the Acceptance, Effectiveness & Role domain scores was significant (*t* = 2.28, *p* = 0.02), effect size (Cohen's *d* = 0.45). This indicates that men scored higher than women.

#### 3.8.3 The Effect of Education Level on Domain Scores of the Attitude Questionnaire

Independent *t*‐test analysis showed no significant differences between the graduated and post‐graduated participants in the Acceptance, Effectiveness & Role domain questions (*t* = −1.40, *p* = 0.16), the Benefits & Adoption (*t* = −1.36, *p* = 0.17) and Challenges and Barriers (*t* = −1.04, *p* = 0.29) domain scores.

#### 3.8.4 The Effect of Work Experience on Domain Scores of the Attitude Questionnaire

The one‐way ANOVA analysis results showed no significant differences between different ranges of work experience in the Acceptance, Effectiveness & Role (F(6132) = 1.38, *p* = 0.22), the Benefits & Adoption (F(6132) = 0.46, *p* = 0.83) and Challenges and Barriers (F(6132) = 1.13, *p* = 0.34) domain scores.

#### 3.8.5 The Effect of TR Experience on Domain Scores of the Attitude Questionnaire

Independent‐samples t‐tests showed no significant differences between participants with and without TR experience in the “Acceptance, Effectiveness, and Role” domain (*t* = 1.46, *p* = 0.14) or the “Benefits and Adoption” domain (*t* = 0.57, *p* = 0.56). The difference in the “Challenges and Barriers” domain did not reach statistical significance (*t* = −1.89, *p* = 0.06).

#### 3.8.6 The Effect of the Service Recipient Group on Domain Scores of the Attitude Questionnaire

The one‐way ANOVA analysis results showed no significant differences between different service recipient groups in the Acceptance, Effectiveness & Role (F(3135) = 0.85, *p* = 0.46), the Benefits & Adoption (F(3135) = 0.96, *p* = 0.41), and challenges and barriers (F(3135) = 1.31, *p* = 0.27) domain scores.

#### 3.8.7 The Effect of the Profession on Domain Scores of the Attitude Questionnaire

Different professional disciplines revealed no statistically significant differences in the reported levels of Acceptance, Effectiveness & Role (F(3135) = 0.63, *p* = 0.59), the Benefits & Adoption (F(3135) = 0.44, *p* = 0.72), and encountered challenges and barriers (F(3135) = 0.1, *p* = 0.95).

#### 3.8.8 The Effect of the Knowledge Level on Domain Scores of the Attitude Questionnaire

The one‐way ANOVA analysis results showed a significant difference between levels of knowledge in the Acceptance, Effectiveness & Role domain scores (F (4134) = 4.42, *p* < 0.01), effect size ($\eta^{2}$ = 0.1). Tukey HSD post‐hoc comparisons showed that level 3 (moderately familiar, rare user) scored significantly higher than level 1 (Very familiar, regular user) (mean difference = 10.79, *p* < 0.01), level 2 (Familiar, occasional user) (mean difference = 8.03, *p* < 0.05), level 4 (Slightly Familiar, never use) (mean difference = 10.08, *p* < 0.01), and level 5 (Not familiar at all and do not use) (mean difference = 11.10, *p* ≤ 0.01). No other pairwise comparisons were statistically significant (*p* > 0.05 in all of them). These differences in the Benefits & Adoption domain were marginally significant (F (4134) = 2.23, *p* = 0.06). There was no significant difference in the challenges and barriers domain score between levels of knowledge (F (4134) = 0.85, *p* = 0.49).

## 4 Discussion

### 4.1 Development and Validation of the Questionnaire

The present study sought to develop a comprehensive questionnaire evaluating the attitudes of rehabilitation professionals regarding TR and to examine how various demographic factors may influence these attitudes. This survey encompassed a diverse group of therapists across multiple disciplines, including speech therapy, physiotherapy, audiology, and occupational therapy. Utilizing an online‐based survey tool, we developed a structured 33‐item questionnaire focusing on three primary domains: Acceptance, Effectiveness & Role, The Benefits & Adoption, and Challenges and Barriers.

The findings of the study indicated that the newly developed questionnaire possesses acceptable content validity and robust face validity. The overall reliability, as indicated by a Cronbach's alpha of 0.90 for the total score and domain‐specific scores exceeding 0.80, underscores the instrument's high internal consistency.

In the exploratory analysis of items categorized into the three domains, questions relating to acceptability, effectiveness, efficiency, and the contributions of various specialties encompassing evaluation, diagnosis, and intervention were grouped under the domain of Acceptance, Effectiveness & Role. Conversely, the second domain, “The Benefits & Adoption,” included items addressing factors such as technological advancements, knowledge acquisition, and the overcoming of barriers, particularly those related to access to technology in the application of TR, while also acknowledging its advantages. Notably, the classification of the consultation item, which was derived from the contributions of various professionals within the field of TR, deviated from initial expectations. Despite its logical alignment with the components of domain 1, the exploratory analysis indicated its fitting within domain 2. Acknowledging the significance of this topic, the authors made the evaluative decision to retain this item within the second domain. However, it is suggested that this item be included in further studies with this questionnaire in the domain of Acceptance, Effectiveness & Role (factor loading in this domain was 0.28).

The third domain, Challenges and Barriers, unearthed a multitude of concerns, including issues related to internet access, associated costs, inadequate knowledge among both professionals and clients, potential reductions in service quality, and privacy‐related apprehensions.

In the CFA, all fit indices except for the TLI were found to fall within acceptable ranges. The TLI value of 0.83 was slightly below the commonly recommended threshold of 0.90. However, as noted by Hu and Bentler (1999), such cutoff values are generally based on intuition and experience rather than strict statistical justification [32]. Given the adequacy of the other indices (e.g., RMSEA = 0.078, PNFI = 0.64, PCFI = 0.75), this deviation was considered acceptable. It is noteworthy that Aloyuni et al. (2020) developed a more limited 14‐item questionnaire that primarily assessed knowledge, attitudes, and barriers toward TR; although content validity was evaluated in their study, the specific CVI and CVR values were not reported [20]. In line with this study, the research conducted by Dadgar et al. (2021) reported CVR and CVI metrics of 0.99, as evaluated by five experts, indicative of an acceptable level for their questionnaire concerning TR during the COVID‐19 pandemic [35]. To the best of our knowledge, no prior study has conducted a structured exploratory factor analysis of a TR attitude questionnaire among diverse rehabilitation professionals. Also, the questionnaire's validity was assessed, including Cronbach's alpha for both the total scale and each domain, which had adequate internal consistency to examine the study's purpose. Although it should be noted that the relatively small sample size (*N* = 139) in relation to the number of items [36] and the low response rate (~28%) should be explicitly acknowledged and considered when interpreting the findings. Although all respondents were experts in the relevant field, enhancing the quality and relevance of the data, the low participation may partly reflect limited familiarity with the concept or its limited application in the study context, despite the use of response incentives. These factors may affect the generalizability of the results and warrant cautious interpretation. Nevertheless, despite the limited sample size, factor analysis was successfully conducted during model validation, and most goodness‐of‐fit indices supported the adequacy of the model, although the TLI value showed a slight deviation from the recommended threshold.

### 4.2 Investigating Attitudes of Rehabilitation Professionals Toward Telerehabilitation

The present study findings showed that the majority of professionals rated TR's acceptability and effectiveness as moderate (e.g., scores of 2 or 3 on the 1‐to‐5 scale) rather than as completely acceptable or completely effective, from both expert and general population perspectives. This suggests a cautious attitude and indicates a need for further research and development to enhance TR's appeal and efficacy. Most experts in this study believed that the adoption of TR was less acceptable and efficient from the public's perspective than from the professional standpoint. However, Rahimi et al.'s study (2024) indicated that, despite this belief among experts, patients view the lack of access to TR as a significant challenge, especially during the pandemic [37]. This gap between professional perception and patient needs is further addressed in recent literature, which suggests that TR is increasingly recognized as a viable alternative or a necessary complement to traditional care, with systematic reviews showing comparable satisfaction levels and similar functional outcomes across various settings. Specifically, evidence from systematic reviews on real‐time video telerehabilitation confirms that this modality can achieve satisfaction and adherence rates that are equal to, or in some cases better than, conventional in‐person physiotherapy [38]. Despite this current caution, our findings suggest that TR holds significant promise for rehabilitation, particularly in speech therapy. This is likely due to the nature of speech therapy and its lesser reliance on physical tools, making it more adaptable to remote settings [39]. Furthermore, Professionals recognize the potential of TR across various rehabilitation disciplines. From the point of view of the majority of experts, TR was most used in the field of counseling, although according to them, it was also used in the fields of evaluation to intervention. These results could be due to the fact that some rehabilitation disciplines depend on equipment for evaluation, diagnosis, and treatment. This pattern of hesitation is particularly evident in multidisciplinary rehabilitation; while clinicians may acknowledge the theoretical advantages of remote services, they often remain cautious due to discipline‐specific concerns regarding the quality of clinical assessment, the maintenance of therapeutic rapport, and ensuring patient safety in a digital environment. Moreover, the successful adoption of these models remains heavily influenced by broader contextual factors, including staff readiness, existing technological infrastructure, clinical workload, and the perceived loss of physical contact, all of which continue to shape how telerehabilitation is integrated into practice [40].

This study identified limitations, particularly in communication modalities. Although video‐based interactions were viewed as superior to text‐based communication, the lower scores for text‐based interactions highlight the need to explore innovative text‐based interventions to improve accessibility and inclusivity. This result is in line with Özden's study (2022), which stated that Video TR offers clear benefits compared to traditional text‐based approaches in multiple therapeutic settings [41].

In contrast to the current acceptability ratings, the specialists exhibited a strong positive outlook on the role of technological advancements in shaping the future of TR. This optimism extends to the belief that increased knowledge and the removal of barriers like internet access will contribute significantly to wider TR adoption. Interestingly, while some participants predicted a gradual shift towards widespread TR use, the majority believed that increased knowledge and reduced barriers will play a more crucial role in its widespread implementation. In this regard, a systematic review study suggested that the widespread implementation of TR is significantly influenced by knowledge increased and reduced barriers [42]. This is consistent with broader systematic evidence suggesting that the successful uptake of such interventions, like cardiac telerehabilitation, is not solely dependent on individual clinician attitudes but is significantly driven by overcoming system‐level barriers and providing robust institutional support [43]. The study highlights a shared belief among participants that TR can significantly benefit individuals with limited access to traditional rehabilitation services. This emphasizes the potential of TR to bridge healthcare disparities and ensure equitable access to essential care. The findings elicit the significant insights into the perceptions and experiences of rehabilitation specialists regarding TR, representing a vital advancement in understanding how professionals view and are prepared for the integration of technology into their practice. This study elucidates both the potential benefits and the challenges that must be navigated for effective implementation. In conclusion, while current acceptability is moderate, results from this study indicate a generally positive attitude toward the future potential of TR among rehabilitation specialists, particularly concerning the perceived roles and benefits associated with TR for patient care and service delivery. In this line, Suso‐Martí et al (2021) in an Umbrella and Mapping Review with Meta–Meta‐Analysis showed that telerehabilitation offers positive clinical results, even comparable to conventional face‐to‐face rehabilitation approaches [44].

A considerable majority of participants expressed favorable opinions regarding the impact of technological advancements. This aligns with similar surveys conducted in other countries, including studies from Canadian, Arabic, Korean, and Iranian contexts [45]. For instance, Cho et al. (2023) reported positive attitudes among physical and occupational therapists toward telerehabilitation, revealing that participants with prior telerehabilitation experience exhibited a more favorable disposition toward its use, thus underscoring the influence of experiential factors on future intentions to adopt telerehabilitation [21]. Saeed et al.'s (2024) study, despite optimism about the potential of TR to increase access and convenience of care, there were concerns about maintaining patient follow‐up, privacy, and the varying healthcare providers in deploying technological tools effectively. One of the notable positive sentiments among professionals revolved around the potential benefits of TR in terms of reducing service delivery time and costs, improving access to care, and extending the reach of rehabilitation services [46].

Despite the positive views, professionals expressed concerns regarding challenges and barriers to the widespread adoption of TR, including lack of access to technology, concerns about confidentiality, and the anticipated deterioration in quality of rehabilitation services. Addressing these challenges will be crucial for successful TR deployment in rehabilitation settings. In Cho's questionnaire on barriers to TR, there were questions about insufficient insurance costs, privacy security, and lack of technical support, and interestingly, therapists with TR experience had higher scores on lack of technical support, meaning that they are more concerned about this issue, although this was not statistically significant [21].

Statistical analyses showed that age, gender, education level, work experience, and the type of service‐recipient group did not significantly affect participants' attitudes in any domain, except for a gender‐related difference in the “Acceptance, Effectiveness, and Role” domain. The lack of association with general work experience may reflect the novelty of TR in some countries, where its structured integration remains relatively recent; indeed, the previous TR‐related article was published during the COVID‐19 era in 2021 [35]. However, actual experience with TR significantly influenced participants' overall attitudes. This finding may indicate that appreciation for TR, along with its associated challenges and benefits, transcends demographic differences and fosters a more cohesive perspective among rehabilitation specialists. In contrast, the significant gender difference in the “Acceptance, Effectiveness, and Role” domain warrants further investigation, as it may shed light on how attitudes toward rehabilitation technology are shaped by individual experiences and professional perspectives. Conversely, a significant difference in the Acceptance, Effectiveness & Role domain based on gender warrants further investigation. Understanding underlying reasons for this gender disparity could yield valuable insights into how attitudes toward technology in rehabilitation are nuanced by individual experiences and perspectives. The study revealed a notable difference in knowledge levels between the Acceptance, Effectiveness & Role scores. These findings indicate a non‐linear association between knowledge and attitude, suggesting that greater knowledge does not necessarily lead to more positive attitudes, as the most favorable scores were observed only in one intermediate knowledge group within the Acceptance, Effectiveness & Role domain. As TR continues to evolve, investment in educational initiatives that emphasize its benefits and practical applications may help reduce resistance and enhance the overall effectiveness of TR interventions. Moreover, the absence of significant differences among professional groups suggests that attitudes toward TR may be broadly similar across rehabilitation disciplines, reflecting a shared perspective on its role and challenges. Consequently, it appears that simply increasing theoretical knowledge may not be sufficient to change professional perspectives; instead, the transition to routine clinical use likely requires a combination of structured training, sustained institutional support, and direct practical exposure to telerehabilitation tools [47].

### 4.3 Implications for Practice and Future Research

The implications of these findings are important for rehabilitation practice. While recent reviews confirm that telerehabilitation can be effective across diverse domains, its ultimate success depends on more than just professional enthusiasm. A sustainable implementation requires a foundation of structured training, reliable technology, and strong institutional backing. This necessity is further emphasized by scoping reviews on the factors facilitating or inhibiting TR, which suggest that moving toward “standard care” requires stakeholders to prioritize supportive organizational frameworks. Furthermore, the potential of these digital interventions is particularly evident for specialized groups, as guided telerehabilitation has been shown to significantly improve functional performance in community‐dwelling older adults [48].

The insights derived from this study possess substantial implications for the training and integration of TR practices within rehabilitation disciplines. Given the positive attitudes toward TR, along with identified challenges, stakeholders in rehabilitation must prioritize the development of supportive frameworks, including targeted training programs, technological infrastructure enhancement, and policies that promote the effective incorporation of TR into standard care practices. Future research should examine whether positive attitudes translate into actual clinical adoption and whether discipline‐specific barriers differ across audiology, speech therapy, physiotherapy, and occupational therapy. It would also be useful to evaluate blended models of care, because recent evidence suggests that hybrid approaches may help address the limitations of fully remote services while preserving accessibility and convenience. In line with this, recent overviews of systematic reviews suggest that telerehabilitation solutions are most effective when they are thoughtfully embedded within the entire patient pathway, serving as a flexible component of care rather than a mere substitute for face‐to‐face interaction [40].

Furthermore, future research should continue to explore the nuances of specialist attitudes toward TR, expanding the sample size and incorporating more diverse professional backgrounds. Longitudinal studies that track changes in attitudes over time and correlate them with the actual deployment of TR services will be vital as the field evolves. Additionally, exploring patient perspectives on TR, particularly concerning rehabilitation specialists' views, is essential for creating a holistic understanding of the impact of TR on care delivery and outcomes.

### 4.4 Limitation

A primary limitation of this study was the relatively small sample size. Although the survey was initially distributed to 486 specialists, only 139 participants returned completed questionnaires. While this sample size was sufficient for conducting an EFA, it posed constraints for subsequent analyses. Specifically, due to these sample size limitations, both the EFA and CFA were conducted on the same dataset. Although this approach allowed for the examination of internal consistency and model fit, it precluded independent cross‐validation. Consequently, the findings should be interpreted as providing only preliminary support for the instrument's structure, and further research involving larger, independent samples is required to confirm the generalizability of these results

Another limitation of this study was the combined assessment of knowledge and usage frequency regarding TR. Future research could address these dimensions separately for greater precision. This study analyzed age and work experience as grouped variables, potentially masking subtle variations. Future research should treat these as continuous predictors (e.g., regression models) for greater precision. Numerical scores were standardized for clarity but may need careful interpretation.

The categorization of service recipient groups combined mutually exclusive age‐specific categories (children, adults, and elderly) with an inclusive “all groups” category. This conceptual overlap limited direct comparability between groups in ANOVA testing. Future studies should employ factorial designs analyzing service to each age group as independent binary variables (served/not served) or use mutually exclusive classification schemes.

Finally, the questions selected for designing this questionnaire aimed to be suitable for posing across all rehabilitation domains and to create a general questionnaire. Consequently, specific questions pertinent to particular disciplines may not have been included.

## 5 Conclusion

This study developed and validated a questionnaire with satisfactory psychometric properties for assessing rehabilitation professionals' attitudes toward telerehabilitation. Overall, rehabilitation professionals reported positive attitudes toward telerehabilitation, particularly regarding its potential to expand access to services across wider geographical areas and support service delivery during pandemics. At the same time, limited internet access and concerns about service quality were identified as important barriers to its utilization. The findings also suggest that the relationship between familiarity with telerehabilitation and attitudes may not be uniformly linear within the Acceptance, Effectiveness, and Role domain. Although further validation in larger and more diverse samples is warranted, the developed questionnaire provides a comprehensive tool for evaluating attitudes toward telerehabilitation and may facilitate future research on factors influencing its adoption among rehabilitation professionals.

## Author Contributions

**Seyede Zohre:** conceptualization, writing – review and editing, writing – original draft, investigation. **Elham Tavanai:** conceptualization, investigation, data curation. **Fatemeh Salmani:** methodology, validation, software, formal analysis. **Mahdieh Karimi:** data curation, investigation. **Vida Rahimi:** conceptualization, investigation, methodology, validation, supervision, writing – review and editing, writing – original draft.

## Funding

The authors have nothing to report.

## Ethics Statement

This study has been approved with code: IR.TUMS.FNM.REC.1402.032 in the Ethics Committees of the University of Medical Sciences.

## Conflicts of Interest

The authors declare no conflicts of interest.

## Transparency Statement

The lead author, Vida Rahimi, affirms that this manuscript is an honest, accurate, and transparent account of the study being reported; that no important aspects of the study have been omitted; and that any discrepancies from the study as planned (and, if relevant, registered) have been explained.

## Supporting information


Supporting File

## Data Availability

The data that support the findings of this study are available on request from the corresponding author. The data are not publicly available due to privacy or ethical restrictions.

## References

[1] R. Wootton , N. G. Patil , R. E. Scott , and K. Ho , ed., Telehealth in the Developing World (Royal Society of Medicine Press; Ottawa (ON): International Development Research Centre, 2009).

[2] R. Ravi , D. R. Gunjawate , K. Yerraguntla , and C. Driscoll , “Knowledge and Perceptions of Teleaudiology Among Audiologists: A Systematic Review,” Journal of Audiology & Otology 22, no. 3 (2018): 120–127, 10.7874/jao.2017.00353. 29719949 PMC6103494

[3] W. Swanepoel de , J. L. Clark , D. Koekemoer , et al., “Telehealth in Audiology: The Need and Potential to Reach Underserved Communities,” International Journal of Audiology 49, no. 3 (2010): 195–202, 10.3109/14992020903470783. 20151929

[4] N. R. Chumbler , W. C. Mann , S. Wu , A. Schmid , and R. Kobb , “The Association of Home‐Telehealth Use and Care Coordination With Improvement of Functional and Cognitive Functioning in Frail Elderly Men,” Telemedicine Journal and e‐Health: The Official Journal of the American Telemedicine Association 10, no. 2 (2004): 129–137, 10.1089/tmj.2004.10.129. 15319042

[5] J. A. Sanford , H. Hoenig , P. C. Griffiths , T. Butterfield , P. Richardson , and K. Hargraves , “A Comparison of Televideo and Traditional In‐Home Rehabilitation in Mobility Impaired Older Adults,” Physical & Occupational Therapy in Geriatrics 25, no. 3 (2007): 1–18.

[6] J. Craig and V. Petterson , “Introduction to the Practice of Telemedicine,” Journal of Telemedicine and Telecare 11, no. 1 (2005): 3–9, 10.1177/1357633X0501100102. 15829036

[7] S. Ryu , “Telemedicine: Opportunities and Developments in Member States: Report on the Second Global Survey on ehealth 2009 (Global Observatory for Ehealth Series, Volume 2),” Healthcare Informatics Research 18, no. 2 (2012): 153.

[8] S. Movahedazarhouligh , R. Vameghi , N. Hatamizadeh , E. Bakhshi , and S. M. Moosavy Khatat , “Feasibility of Telerehabilitation Implementation as a Novel Experience in Rehabilitation Academic Centers and Affiliated Clinics in Tehran: Assessment of Rehabilitation Professionals' Attitudes,” International Journal of Telemedicine and Applications 2015, no. 1 (2015): 468560, 10.1155/2015/468560. 26640483 PMC4660026

[9] O. World Health , Telemedicine: Opportunities and Developments in Member states. Report on the Second Global Survey on eHealth (World Health Organization, 2010).

[10] E. R. Cohn and J. Cason , “Telepractice, Telehealth, and Telemedicine: Acquiring Knowledge From Other Disciplines,” Perspectives of the ASHA Special Interest Groups 1, no. 18 (2016): 19–29.

[11] T.‐H. Peng , J. J. Eng , A. Harris , et al., “A Survey of the Experiences of Delivering Physiotherapy Services Through Telerehabilitation During the COVID‐19 Pandemic,” Frontiers in Rehabilitation Sciences 5 (2024): 1486801, 10.3389/fresc.2024.1486801. 39512760 PMC11540677

[12] G. H. Saunders and A. Roughley , “Audiology in the Time of COVID‐19: Practices and Opinions of Audiologists in the UK,” International Journal of Audiology 60, no. 4 (2021): 255–262, 10.1080/14992027.2020.1814432. 32909474

[13] M. Ce , B. Ta , S. Ct , et al., “Awareness, Attitude and Expectations of Physiotherapy Students on Telerehabilitation,” Medical Science Educator 31, no. 2 (2021): 627–636, 10.1007/s40670-021-01234-w. 33619445 PMC7889412

[14] S. Ullah , A. M. Maghazil , A. Z. Qureshi , S. Tantawy , I. S. Moukais , and A. A. Aldajani , “Knowledge and Attitudes of Rehabilitation Professional Toward Telerehabilitation in Saudi Arabia: A Cross‐Sectional Survey,” Telemedicine and e‐Health 27, no. 5 (2021): 587–591, 10.1089/tmj.2020.0016. 32384256

[15] N. N. M R and J. Seethapathy , “Tele‐Audiology in India: Current and Future Trends in Knowledge, Attitude, and Practice Among Audiologists,” Journal of Audiology and Otology 26, no. 3 (2022): 130–141, 10.7874/jao.2021.00584. 35538867 PMC9271733

[16] Z. Zahid , S. Atique , M. H. Saghir , I. Ali , A. Shahid , and R. A. Malik , “A Commentary on Telerehabilitation Services in Pakistan: Current Trends and Future Possibilities,” International Journal of Telerehabilitation 9, no. 1 (2017): 71–76, 10.5195/ijt.2017.6224. 28814996 PMC5546563

[17] B. Mui , M. Lawless , B. H. B. Timmer , et al., “Australian Hearing Healthcare Stakeholders' Experiences of and Attitudes Towards Teleaudiology Uptake: A Qualitative Study,” Speech, Language and Hearing 28, no. 1 (2025): 1–10.

[18] B. Parmar , E. Beukes , and S. Rajasingam , “The Impact of COVID‐19 on Provision of UK Audiology Services & On Attitudes Towards Delivery of Telehealth Services,” International Journal of Audiology 61, no. 3 (2022): 228–238, 10.1080/14992027.2021.1921292. 34010078

[19] G. Bajaj and S. Karuppali , “Knowledge, Attitudes, and Practices of Speech Language Pathologists in India About Telerehabilitation Services During the COVID‐19 Pandemic,” Codas 34, no. 6 (2022): e20210193, 10.1590/2317-1782/20212021193. 35584517 PMC9886298

[20] S. Aloyuni , R. Alharbi , F. Kashoo , et al., “Knowledge, Attitude, and Barriers to Telerehabilitation‐Based Physical Therapy Practice in Saudi Arabia,” Healthcare 8, no. 4 (2020): 460, 10.3390/healthcare8040460. 33158298 PMC7711516

[21] H.‐M. Cho , H. Kim , J. Jang , et al., “Attitude Toward Telerehabilitation Among Physical and Occupational Therapists in Korea: A Brief Descriptive Report,” Brain & NeuroRehabilitation 16, no. 1 (2023): 8, 10.12786/bn.2023.16.e8. PMC10079478 37033001

[22] S. Movahedazarhouligh , R. Vameghi , N. Hatamizadeh , E. Bakhshi , and S. M. Mousavi Khatat , “The Level of Awareness of Rehabilitation Professionals Employed in Rehabilitation Academic Centers Regarding Tele‐Rehabilitation Technology,” Iranian Rehabilitation Journal 13, no. 2 (2015): 57–61. 10.1155/2015/468560 PMC4660026 26640483

[23] Y. Kurtaiş Aytür , “Prerequisites and Barriers to Telerehabilitation in Patients With Neurological Conditions: A Narrative Review,” NeuroRehabilitation: An International, Interdisciplinary Journal 56, no. 1 (2025): 61–71, 10.3233/NRE-240092. 39269858

[24] L. B. Mokkink , C. B. Terwee , D. L. Patrick , et al., “The COSMIN Checklist for Assessing the Methodological Quality of Studies on Measurement Properties of Health Status Measurement Instruments: An International Delphi Study,” Quality of Life Research 19 (2010): 539–549, 10.1007/s11136-010-9606-8. 20169472 PMC2852520

[25] P. A. Stehr‐Green , J. K. Stehr‐Green , A. Nelson , L. Alexander , G. C. Mejia , and P. D. MacDonald , “Developing a Questionnaire,” FOCUS Field Epidemiology 2, no. 2 (2003): 1–6.

[26] R. F. DeVellis and C. T. Thorpe , Scale Development: Theory and Applications (Sage Publications, 2021).

[27] C. H. Lawshe , “A Quantitative Approach to Content Validity,” Personnel Psychology/Berrett‐Koehler Publishers 28 (1975): 563–575.

[28] M. Romero Jeldres , E. Díaz Costa , and T. Faouzi Nadim , “Editors. A Review of Lawshe's Method for Calculating Content Validity in the Social Sciences,” Frontiers Media SA 8 (2023): 1271335.

[29] D. F. Polit , C. T. Beck , and S. V. Owen , “Is the CVI an Acceptable Indicator of Content Validity? Appraisal and Recommendations,” Research in Nursing & Health 30, no. 4 (2007): 459–467, 10.1002/nur.20199. 17654487

[30] J. Hair , R. Andreson , R. Tatham , and W. Black , Multivariate Data Analysis (Prentice‐Hall Inc., United States of America, 1998). 5th (ed).

[31] S. B. Plichta , E. A. Kelvin , and B. H. Munro , Munro's Statistical Methods for Health Care Research (Wolters Kluwer Health, Lippincott Williams & Wilkins, 2013). 6th ed..

[32] L. Hu and P. M. Bentler , “Cutoff Criteria for Fit Indexes in Covariance Structure Analysis: Conventional Criteria Versus New Alternatives,” Structural Equation Modeling: A Multidisciplinary Journal 6, no. 1 (1999): 1–55.

[33] J. C. Nunnally and I. H. Bernstein , Psychometric Theory (McGraw‐Hill, 1994). 3rd ed..

[34] R. B. Kline , Principles and Practice of Structural Equation Modeling (Guilford Press, 2016). 4th ed..

[35] H. Dadgar , S. Maroufizadeh , J. Bakhtiyari , and A. Vosoughi , “Feasibility, Satisfaction With, and Attitude Toward Telerehabilitation During COVID‐19 Pandemic: Experience of Iranian Rehabilitation Professionals,” Iranian Rehabilitation Journal 19, no. 4 (2021): 399–406.

[36] H. W. Marsh and D. Grayson , “Latent Variable Models of Multitrait‐Multimethod Data,” in Structural equation modeling: Concepts, issues, and applicationsed, ed. R. H. Hoyle (Sage Publications, Inc, 1995), 177–198.

[37] V. Rahimi , E. Tavanai , S. M. R. Taghavi , and M. E. Khalili , “Uncovering the Effects of Pandemic Conditions on Hearing Aid Experiences: A Dual Perspective From Audiologists and Hearing Aid Users,” Disability and Rehabilitation. Assistive Technology 20, no. 3 (2025): 652–662, 10.1080/17483107.2024.2405557. 39374325

[38] J. Simmich , M. H. Ross , and T. Russell , “Real‐Time Video Telerehabilitation Shows Comparable Satisfaction and Similar or Better Attendance and Adherence Compared With In‐Person Physiotherapy: A Systematic Review,” Journal of Physiotherapy 70, no. 3 (2024): 181–192, 10.1016/j.jphys.2024.06.001. 38879432

[39] D. Brennan , A. Georgeadis , and C. Baron , “Telerehabilitation Tools for the Provision of Remote Speech‐Language Treatment,” Topics in Stroke Rehabilitation 8, no. 4 (2002): 71–78, 10.1310/U7KV-DY7U-Q6QP-LVBP. 14523731

[40] B. Nicolas , E. Leblong , B. Fraudet , P. Gallien , and P. Piette , “Telerehabilitation Solutions in Patient Pathways: An Overview of Systematic Reviews,” Digital Health 10 (2024): 1–13, 10.1177/20552076241294110. PMC11536572 39502480

[41] F. Özden , Z. Sarı , Ö. N. Karaman , and H. Aydoğmuş , “The Effect of Video Exercise‐Based Telerehabilitation on Clinical Outcomes, Expectation, Satisfaction, and Motivation in Patients With Chronic Low Back Pain,” Irish Journal of Medical Science 191, no. 3 (2022): 1229–1239, 10.1007/s11845-021-02727-8. 34357527

[42] N. Rabanifar and K. Abdi , “Barriers and Challenges of Implementing Telerehabilitation: A Systematic Review,” Iranian Rehabilitation Journal 19, no. 2 (2021): 121–128.

[43] D. Ferrel‐Yui , D. Candelaria , T. R. Pettersen , R. Gallagher , and W. Shi , “Uptake and Implementation of Cardiac Telerehabilitation: A Systematic Review of Provider and System Barriers and Enablers,” International Journal of Medical Informatics 184 (2024): 105346, 10.1016/j.ijmedinf.2024.105346. 38281451

[44] L. Suso‐Martí , R. La Touche , A. Herranz‐Gómez , S. Angulo‐Díaz‐Parreño , A. Paris‐Alemany , and F. Cuenca‐Martínez , “Effectiveness of Telerehabilitation in Physical Therapist Practice: An Umbrella and Mapping Review With Meta–Meta‐Analysis,” Physical Therapy 101, no. 5 (2021): pzab075, 10.1093/ptj/pzab075. 33611598 PMC7928612

[45] E. Giesbrecht , M. E. Major , M. Fricke , et al., “Telerehabilitation Delivery in Canada and the Netherlands: Results of a Survey Study,” JMIR Rehabilitation and Assistive Technologies 10, no. 1 (2023): e45448, 10.2196/45448. 36806194 PMC9989917

[46] W. Saeed , N. Asif , M. Malik , et al., “Knowledge, Attitude and Skills of Physiotherapists Towards Tele‐Rehabilitation,” Journal of Health and Rehabilitation Research 4, no. 2 (2024): 43–48.

[47] S. Stampa , C. Thienel , P. Tokgöz , O. Razum , and C. Dockweiler , “Factors Facilitating and Inhibiting the Implementation of Telerehabilitation—A Scoping Review,” Healthcare 12, no. 6 (2024): 619, 10.3390/healthcare12060619. 38540583 PMC10970368

[48] C. Gamble , J. van Haastregt , E. van Dam van Isselt , S. Zwakhalen , and J. Schols , “Effectiveness of Guided Telerehabilitation on Functional Performance in Community‐Dwelling Older Adults: A Systematic Review,” Clinical Rehabilitation 38, no. 4 (2024): 457–477, 10.1177/02692155231217411. 38013415 PMC10898211

