# Supplementary material for: Exploring Rehabilitation Professionals' Attitudes Toward Telerehabilitation Using Developing a New Questionnaire: A Cross ‐Sectional Study
Source: Health Sci Rep. 2026 Aug 31;9(9):e73121. doi: 10.1002/hsr2.73121 (PMC13530384; doi:10.1002/hsr2.73121)
Supplement: Supplementary file 1 [file HSR2-9-e73121-s001.docx]

Supplementary 1: Exploratory factor analysis of 33 items of questionnaire

|  | **Acceptance, Effectiveness & Role** | **Benefits & Adoption** | Challenges and Barriers |
| --- | --- | --- | --- |
| Q1 | 0.596 |  |  |
| Q2 | 0.815 |  |  |
| Q3 | 0.619 |  |  |
| Q4 | 0.794 |  |  |
| Q5 | 0.332 |  |  |
| Q6 | 0.419 |  |  |
| Q7 | 0.387 |  |  |
| Q8 | 0.373 |  |  |
| Q9 | 0.579 |  |  |
| Q10 | 0.522 |  |  |
| Q11 | 0.41 | 0.51 |  |
| Q12 | 0.397 |  |  |
| Q13 | 0.425 |  |  |
| Q14 | 0.587 |  |  |
| Q15 | 0.28 | 0.52 |  |
| Q16 |  | 0.769 |  |
| Q17 |  | 0.826 |  |
| Q18 |  | 0.841 |  |
| Q19 |  | 0.666 |  |
| Q20 |  | 0.450 |  |
| Q21 |  | 0.325 |  |
| Q22 |  | 0.539 |  |
| Q23 |  | 0.562 |  |
| Q24 |  | 0.521 |  |
| Q25 |  |  | 0.408 |
| Q26 |  |  | 0.442 |
| Q27 |  |  | 0.414 |
| Q28 |  |  | 0.451 |
| Q29 |  |  | 0.569 |
| Q30 |  |  | 0.697 |
| Q31 |  |  | 0.515 |
| Q32 |  |  | 0.797 |
| Q33 |  |  | 0.685 |

Supplementary 2. Distribution of response to attitude to TR among rehabilitation specialist

|  |  | **Distribution of response to attitude to TR among rehabilitation professionals** | | | | | | |
| --- | --- | --- | --- | --- | --- | --- | --- | --- |
| **Domains** | **Question** |  | | **1** | **2** | **3** | **4** | **5** |
| Acceptance, Effectiveness & Role | Q1 | From a professional perspective, how acceptable is telerehabilitation in your country? | | 12.2% | 25.9% | 46.8% | 10.1% | 5% |
|  | Q2 | From a general public perspective, how acceptable is telerehabilitation in your country? | | 21.6% | 35.3% | 27.3% | 12.2% | 3.6% |
|  | Q3 | From a professional perspective, how effective is telerehabilitation in your country? | | 6.8% | 21.6% | 40.3% | 23% | 6.5% |
|  | Q4 | From a general public perspective, how effective is telerehabilitation in your country? | | %23 | %35.3 | %25.9 | 12.2% | 3.6% |
|  | Q5 | In your opinion, how effective is video communication for telerehabilitation? | | 2.2% | 5.8% | 19.4% | 33.1% | 39.6% |
|  | Q6 | In your opinion, how effective is audio communication for telerehabilitation? | | 6.5% | 19.4% | 25.2% | 33.8% | 15.5% |
|  | Q7 | In your opinion, how effective is text-based communication for telerehabilitation? | | 6.5% | 27.3% | 28.8% | 16.5% | 10.8% |
|  | Q8 | In your opinion, what is the role of audiology in telerehabilitation services? | | 19.4% | 27.3% | 28.1% | 20.9% | 4.3% |
|  | Q9 | In your opinion, what is the role of physiotherapy in telerehabilitation services? | | 13.7% | 18.7% | 30.2% | 25.2% | 12.2% |
|  | Q10 | In your opinion, what is the role of occupational therapy in telerehabilitation services? | | 10.8% | 18% | 27.3% | 30.9% | 12.9% |
|  | Q11 | In your opinion, what is the role of speech therapy in telerehabilitation services? | | 3.6% | 8.6% | 18% | 43.2% | 26.6% |
|  | Q12 | What is the role of telerehabilitation in the assessment process of rehabilitation? | | 5.8% | 18.7% | 30.2% | 28.8% | 16.5% |
|  | Q13 | What is the role of telerehabilitation in the diagnosis process of rehabilitation? | | 10.8% | 18.7% | 31.7% | 25.2% | 13.7% |
|  | Q14 | What is the role of telerehabilitation in the intervention process of rehabilitation? | | 5% | 15.8% | 38.1% | 28.8% | 12.2% |
| Benefits & Adoption | Q15 | What is the role of telerehabilitation in the consultation process of rehabilitation? | | 2.2% | 1.4% | 10.1% | 28.1% | 58.3% |
|  | Q16 | To what extent do you think technological advancements will influence the likelihood of using telerehabilitation in the future? | | 1.4% | 2.2% | 7.9% | 37.4% | 51.1% |
|  | Q17 | To what extent do you think increased knowledge in this field will affect the likelihood of using telerehabilitation in the future? | | 0.7% | 4.3% | 12.9% | 33.8% | 48.2% |
|  | Q18 | To what extent do you think addressing barriers and challenges in telerehabilitation (access to the internet, information technologies like smartphones, etc.) will affect the likelihood of using telerehabilitation in the future? | | 0.7% | 2.2% | 12.9% | 42.4% | 41.7% |
|  | Q19 | The passage of time has no impact on use of telerehabilitation. | | 1.4% | 5.8% | 18.7% | 37.4% | 36.7% |
|  | Q20 | What do you think are the benefits of telerehabilitation in each of the following areas? | Reduces service delivery time | 2.9% | 6.5% | 25.9% | 26.6% | 38.1% |
|  | Q21 |  | - Reduces service delivery costs | 6.5% | 10.8% | 18% | 27.3% | 37.4% |
|  | Q22 |  | - Provides the possibility of providing services to wider areas | 0.7% | 0.7% | 8.6% | 28.8% | 61.2% |
|  | Q23 |  | - Provides the possibility of providing services to patients during pandemics | 0.7% | 0.7% | 6.5% | 30.2% | 61.9% |
|  | Q24 |  | - Provides access to service delivery for patients with special needs | 1.4% | 2.9% | 12.9% | 36.7% | 46% |
| Challenges and **Barriers** | Q25 | What are the contributing factors to the underutilization of telerehabilitation, and to what extent does each factor play a role? | - Lack of internet access or limited access by specialist or patient | 5% | 7.2% | 18.7% | 34.5% | 34.5% |
|  | Q26 |  | - Lack of access or limited access to information and communication technologies (such as computers, tablets, smartphones) by specialist or patient | 5% | 5% | 24.5% | 35.3% | 30.2% |
|  | Q27 |  | - Cost | 6.5% | 21.6% | 33.1% | 24.5% | 14.4% |
|  | Q28 |  | - Lack of knowledge of specialists and patients | 3.6% | 10.8% | 29.5% | 32.4% | 23.7% |
|  | Q29 |  | - Reduction in service quality | 2.9% | 14.4% | 26.6% | 29.5% | 26.6% |
|  | Q30 |  | - Risk that my job might become automated (patients not attending in person) | 8.6% | 12.2% | 33.8% | 22.3% | 23% |
|  | Q31 |  | - Lack of assurance of information confidentiality from the patient's perspective | 10.5% | 15.8% | 37.4% | 25.2% | 10.8% |
|  | Q32 |  | - Undervaluing the services provided from the patient's perspective | 2.9% | 10.1% | 28.1% | 31.7% | 27.3% |
|  | Q33 |  | Mistrust of Technology in Patients | 1.4% | 8.6% | 41.7% | 27.3% | 20.0% |
